# Teachers’ multicultural attitudes and perspective taking abilities as factors in culturally responsive teaching

**DOI:** 10.1111/bjep.12328

**Published:** 2019-12-09

**Authors:** Ceren Su Abacioglu, Monique Volman, Agneta H. Fischer

**Affiliations:** ^1^ Research Institute of Child Development and Education Educational Sciences University of Amsterdam The Netherlands; ^2^ Department of Psychology Social Psychology University of Amsterdam The Netherlands

**Keywords:** culturally responsive teaching, perspective taking, multicultural attitudes and awareness, multicultural education, quantitative research

## Abstract

**Background:**

Culturally responsive teaching (CRT) has been associated with increased student engagement and achievement. Its practice in classrooms, however, has been shown to be less than optimal. Nonetheless, certain teacher qualities have been suggested to facilitate its practice.

**Aims:**

The current study sought quantitative evidence in support of two of these teacher qualities, namely teachers’ multicultural attitudes, and their perspective taking abilities. By identifying the strength of the suggested relationships, we aimed to examine the generalizability of previous findings in the literature and inform teachers’ professional development and interventions.

**Sample:**

A total of 143 primary school teachers from different parts of the Netherlands responded to our online survey.

**Methods:**

We conducted a multivariate multiple regression analysis to investigate the relationship between these qualities and teachers’ engagement in two separate but related components of CRT (i.e., socially sensitive and culturally sensitive teaching).

**Results:**

Results of our analysis yielded significant relationships between the two teacher qualities and the frequency with which teachers engage in socially and culturally sensitive teaching. Perspective taking was a stronger predictor for both aspects of CRT.

**Conclusion:**

These findings signal the significance of incorporating especially perspective taking experiences and exercises into teacher education and professional development programmes, which could benefit all students regardless of their backgrounds. Our results are promising as these qualities are malleable and thus can be improved.

## Background

The debate around diversity currently is a salient and permanent aspect of educational discourse, as learning and teaching in multicultural classrooms have brought major challenges to both teachers and students. The educational position of students with a migration history still continues to be disadvantaged compared to their peers with no history of migration (OECD, 2016). These findings suggest that more attention should be paid to factors that may support students’ educational success (Phalet, Andriessen, & Lens, 2004).

In general, students feel valued, more capable of learning, and more engaged with the learning environment and materials when the teacher is responsive to their needs (e.g., Gay, 2010; Nieto, 2004). Culturally responsive teaching (CRT), defined by Gay (2010, p. 31) as ‘using the cultural knowledge, prior experiences, frames of reference, and performance styles of ethnically diverse students to make learning encounters more relevant to and effective for them’, has been particularly associated with increased engagement and interest in school and increased educational achievement of minoritized students1We use the adjective ‘minoritized’ rather than ‘minority’ when referring to students with a migration history. We believe this better reflects the continued lack of acknowledgment of varying experiences and needs of students who come from historically marginalized racial and ethnic groups, even when they are not a numerical minority in the classroom. (Aronson & Laughter, 2016). While there is a plethora of research on how to improve CRT, its practice in the classrooms has been shown to be less than optimal (Lim, Tan, & Saito, 2019). One explanation for this problem could be that certain teacher qualities are necessary for effective CRT (Gay, 2013).

The current study aims to contribute quantitative evidence to the existing literature by examining teacher qualities that have previously been suggested to be essential for CRT (reviewed in Rychly & Graves, 2012). More specifically, we investigate teachers’ *perspective taking* abilities and their *multicultural attitudes* in relation to their self‐reported CRT. To the best of our knowledge, the current study is the first to examine these connections quantitatively. With this quantitative evidence, we can examine the generalizability of previous findings in the literature, using a larger sample and more robust data. Additionally, by examining the strength of the suggested relationships, we hope to gain more insight in teachers’ professional development and most effective interventions.

### Culturally responsive teaching

The unfavourable educational position of ethnically minoritized students has been attributed to a mismatch between home and school cultures (Phalet *et al.*, 2004). Advocates of CRT have therefore argued that academic knowledge and skills should be connected to students’ personal experiences and frames of reference within a supportive and cooperative environment. This way, learning becomes more meaningful and engaging (Gay, 2000, 2002). Indeed, different aspects of CRT have been found to be related to positive student outcomes, such as increased student engagement, better achievement, and more positive peer relationships.

As detailed by Gay (2002), CRT includes developing a culturally diverse knowledge base by learning about differences in communication and learning styles, and attending to unique cultural qualities of the students and their realities (e.g., racism and discrimination). In order to build this knowledge base, teachers need to learn about the various elements of students’ culture—ranging from tangible culture or family experiences, artefacts, and events to intangible culture such as values, traditions, language, and identity—through their own research and meaningful relationships with students (Morrison, Robbins, & Rose, 2008). This can be accomplished by, for instance, making home visits at the beginning of the school year, giving opportunities to students to share personal experiences via classroom discussions, or asking students to write stories about their lives (Morrison *et al.*, 2008). This would help teachers to identify the ways in which mainstream schooling and culture may differ from the home culture of certain students, and how their culture and language may contribute to their attitudes and behaviours. Turkish society, for instance, is characterized by generational hierarchy. Accordingly, children’s relationships with authority figures such as their fathers and their teachers are, to a great extent, marked by conformity, whereas taking initiative and posing questions are discouraged (Sunar & Fişek, 2005).

Culturally responsive teaching also implies designing culturally relevant curricula and culturally responsive instructions to make learning more relevant and effective (Gay, 2002). Relating learning materials to students’ personal lives can vary from simply posting a song that shows acknowledgement of their students’ backgrounds (Landsman, 2006) to a more thorough examination of the teaching material in order to ensure that it does not only reflect the mainstream perspectives. Feger (2006), for instance, showed that her students, who were predominantly migrants from the Caribbean and Central and South America, were more engaged in reading, more critical about the reading material, and were able to identify more with the selected texts when she included literature that offered characters and problems similar to her students’ lives. Dimick (2012) also showed that when students in an environmental science class were included in a shared decision‐making process to create school projects relevant to their community, they felt not only academically but also socially and politically empowered.

Lastly, CRT comprises demonstrations of cultural caring, building a learning community, and effective cross‐cultural communication (Gay, 2002). In addition to the challenges of addressing diversity issues within the curriculum, the need to address social competence has been increasing, as this is crucial for student engagement (see, e.g., Self Determination Theory; Deci & Ryan, 1985). Team‐building activities, for example, promote social cohesion and a sense of solidarity. Creating an inclusive social–emotional climate helps students to feel more at ease when they express personal opinions and experiences (Cuseo, 2000). Moreover, Harriott and Martin (2016) reported that cooperative learning opportunities among students who differ in their cultural heritage and achievement levels promote friendship formation, prosocial interactions, acceptance of differences between peers, and support for others’ learning. These opportunities thus may help students from various groups to familiarize with each other, facilitate exchange of cultural information, learn to value diversity, and use the cultural resources of their peers in creative problem‐solving (Johnson & Johnson, 2000).

In sum, various CRT practices may lead to more critical and active learning and better school engagement (see Morrison, Robbins, and Rose's synthesis of research on what CRT 'looks like' in classrooms; 2008).

### Teacher qualities essential for CRT

The aforementioned relationships between different aspects of CRT and positive student outcomes suggest that the educational position of minoritized students could be improved with teachers’ attention to the variability in their students’ experiences and needs. However, notwithstanding the expanding literature on these positive outcomes and the availability of practical information on how to improve educational and pedagogical practices, CRT has been criticized to be either not implemented at all (Kim & Pulido, 2015; Ladson‐Billings, 2014) or implemented at a rather superficial level, such as through celebration of ethnic foods (Sleeter & McLaren, 2009). This suggests that many teachers could further improve their capacities to adapt their teaching to the needs of a diverse student body. With the current research, we will examine whether specific teacher qualities are related to the frequency with which teachers engage in the more meaningful aspects of CRT.

In their review, Rychly and Graves (2012) identified three teacher qualities that are especially important for CRT. First, teachers should be able to take their students’ perspectives. This involves replacing one’s own frame of reference by the other’s perspective, and understanding where their students come from and where they stand, when preparing their educational environment, forming and/or implementing the curriculum and the instructional material (Cooper, 2004; McAllister & Irvine, 2002; Robins, Lindsey, Lindsey, & Terrell, 2006). Second, teachers should develop positive attitudes and beliefs about other cultures, as well as be aware of their own cultural frames of reference (Grant & Asimeng‐Boahene, 2006; Nieto, 2004). Lastly, teachers should have knowledge about cultures that are represented in their classrooms to be able to adjust their teaching accordingly (Rychly & Graves, 2012). In the current study, we test the first two proposed relationships by examining whether teachers’ perspective taking abilities and multicultural attitudes are associated with the frequency with which they engage in CRT.

Perspective taking – the ability to perceive things from a point of view other than one’s own (Moskowitz, 2005, p. 277), has been proposed to be a desirable trait for teachers in multicultural settings. It has been previously associated with appreciation and respect for individuals’ unique experiences, and with flexibility, reduced stereotyping (Galinsky & Moskowitz, 2000), and sensitivity to different cultures (Germain, 1998). Teachers who can take the perspectives of their students are able to better understand their students’ different needs and adapt their instruction and curricula to match these needs (Darling‐Hammond, 2000; McAllister & Irvine, 2002). Teachers who can take others’ perspectives are expected to be more successful in providing unbiased education (Rychly & Graves, 2012). We therefore hypothesized that (H1) teachers who have higher perspective taking abilities will more frequently engage in CRT.

In addition to being able to take others’ perspectives, teachers’ own attitudes and beliefs are suggested to be important for CRT as well. Especially implicit stereotypes and negative attitudes can influence student judgements and contribute to unfavourable educational outcomes of minoritized students (Tobisch & Dresel, 2017). Teachers’ decisions on selecting students for various academic tracks, for instance, have been found to be affected by stereotypical achievement expectations that are activated by as little information as a prototypical name (Tobisch & Dresel, 2017). Teachers cannot effectively engage in CRT, unless they hold positive attitudes towards diversity and are aware of their own, sometimes biased, attitudes and beliefs about other cultures (Nieto, 2004). We use the umbrella term ‘multicultural attitudes’ to reflect ‘teachers’ awareness of, comfort with, and sensitivity to issues of cultural pluralism’, following the definition of Ponterotto, Baluch, Greig, and Rivera, (1998, p. 1003). Teachers with more positive multicultural attitudes consider cultural diversity as an asset and feel more compelled to address issues around diversity in their teaching (Ponterotto *et al.*, 1998). We therefore hypothesized that (H2) teachers who have more positive multicultural attitudes will engage in CRT more frequently.

### The current study

We tested whether the extent of teachers’ CRT practices is associated with (1) teachers’ perspective taking abilities and (2) teachers’ multicultural attitudes. Our target group was primary school teachers. Primary school years are very important in students’ developmental trajectories with long‐term consequences in their academic and social development (Swanson, Cunningham, Youngblood, & Spencer, 2009). In addition, we asked teachers to report on their own ethnic background as well as the concentration of ethnically minoritized students in their classroom, since teachers in these classrooms might be more aware of issues around diversity (Edwards, 2016) and thus might engage more in CRT (Thijs & Verkuyten, 2014). Previous studies have shown that the urgency to give attention to diversity matters is more apparent in schools with higher concentrations of ethnically minoritized children, whereas in schools with fewer ethnically minoritized children, discussing such matters is perceived as less relevant and thus harder to achieve (Agirdag, Merry, & Van Houtte, 2016). Moreover, with increased exposure to a diverse student body, teachers may develop more positive attitudes and more awareness about diversity (Allport, 1954). Accordingly, beginning teachers, for instance, may find dealing with diversity more challenging. We therefore also included teachers’ age and years of teaching experience in our study (van Tartwijk, den Brok, Veldman, & Wubbels, 2009).

## Method

### Participants

Hundred and forty‐three primary school teachers from cities in all regions of the Netherlands responded to an online advertisement targeting our specific sample. Participants received €10 for their participation. One person was excluded on the basis of not attending to the questionnaire (all questions had the same ratings), and eight participants were excluded for not meeting our selection criteria. Moreover, one participant was excluded due to her scores that were multivariate outliers. 86.9% of the remaining sample (*M*_age_ = 38.93, *SD*_age_ = 11.71, 84.7% female) indicated Dutch as their first ethnic affiliation, 19.7% of which also identified with a second ethnic background. 13.1% of the sample did not specify their ethnic backgrounds. The participants were predominantly female and white, as also found in previously published studies conducted in the Netherlands (e.g., Abacioglu *et al.*, 2019; Van Den Bergh, Denessen, Hornstra, Voeten, & Holland, 2010). Our sample demographics mirror the teaching force in the Netherlands, which has been increasing in diversity, but is still fairly homogenous.

### Procedure and design

All the questionnaires were administered in Dutch. In order to ensure correct translations, the English questionnaires were translated from and back‐translated to English (except for the Interpersonal Reactivity Index for which we used an existing translation in Dutch, see the Materials section). Moreover, items were reviewed by a team of seven individuals comprising teacher educators, in‐service teachers, and educational scientists for the appropriateness of the items for the Dutch educational context.

For participant recruitment, we used Facebook’s advertising opportunities to target teachers with the desired background (i.e., primary school in‐service teachers in Dutch schools). The advertisement included minimal information, indicating that we are recruiting for a study on cultural diversity. The study’s duration and the amount of monetary compensation were included in the description.

Ethical approval for this study (2017CDE7604) was granted by the Ethics Review Board of the Faculty of Social and Behavioral Sciences, University of Amsterdam, the Netherlands. The participating teachers filled in an online survey on Qualtrics that lasted about 15 min to complete. Participation was voluntary and anonymous as the survey ended immediately if the participant did not give consent at the beginning of the survey.

### Measures

#### Culturally responsive teaching practices

Teachers responded to 40 statements on a 5‐point Likert‐type scale, about their practices in student assessment, curriculum and instruction, classroom management, and cultural enrichment. The items were based on the Culturally Responsive Teaching Self‐efficacy Scale (CRTSES; Siwatu, 2007), but have been adapted to measure *practices* in the classrooms. An example item from the survey is ‘I *identify* the diverse needs of my students’ (responses on a scale from 1: never to 5: always).

Some items were excluded from our analyses because of the following reasons: they were not representative of the Dutch educational context, they were too subject specific (e.g., ‘I tell about the achievements of culturally different others in Math’), they were about the home life of the students, or they were too similar to other items. For instance, the item ‘I identify ways in which standardized tests can be prejudiced against culturally different students’ does not apply to the Dutch context, because as in the Netherlands a nation‐wide standardized test is used by all schools (i.e., CITO). Individual teachers do not have any control over its content.

Conceptually, we retained items that fell under two categories: items that were representative of teachers’ cultural responsiveness (e.g., ‘I use the cultural background of my students to make learning meaningful’), and an overall responsiveness to students’ academic (e.g., academic strengths and weaknesses of students) and social needs (e.g., positive relationships with classmates). In order to test this categorization, we performed a factor analysis with two forced factors as detailed in the Data Analysis section.2In our data collection, we also included a 3‐item measure of multicultural education pertaining more to prejudice reduction practices (used in e.g., Verkuyten & Thijs, 2002). However, as the items did not show convergent validity with and were not as robust as the CRT measure, we did not include them in further steps. Examining the factor structure of these items indicated a good fit for a two‐factor solution of the data. Throughout the text, these categories are referred to as ‘culturally sensitive teaching’ (α = .83) and ‘socially sensitive teaching’, respectively (α = .73). Sum scores were calculated per category (see the Appendix for the retained items and their factor loadings).

#### Perspective taking

Teachers’ self‐reported perspective taking abilities were measured using the perspective taking subscale of the Dutch version of the Interpersonal Reactivity Index (De Corte *et al.*, 2007), originally developed by Davis (1983). Participants responded to seven items on a 5‐point Likert scale (1: does not describe me well, 5: describes me very well), asking them to report how likely they are to try seeing things from another person’s point of view. An example item from the survey is ‘I sometimes try to understand my friends better by imagining how things look from their perspective’. Sum scores were calculated per participant. Higher scores indicate stronger perspective taking abilities (α = .72).

#### Teacher multicultural attitudes

Teachers’ cultural awareness and sensitivity were assessed with the Teacher Multicultural Attitudes Survey (TMAS; Ponterotto *et al.*, 1998). Teachers responded to 20 statements on a 5‐point Likert scale (1: strongly disagree, 5: strongly agree). An example item from the survey is ‘Teachers have the responsibility to be aware of their students’ cultural backgrounds’. TMAS has shown low social desirability and is unique in its focus on the educational context. It has yielded convergent correlations with scales measuring individuals’ subtle racial and gender bias (e.g., the Quick Discrimination Index; Ponterotto *et al.*, 1995) and attitudes towards and interactions with outgroup members (e.g., the Multigroup Ethnic Identity Measure, Other Group Orientation subscale; Phinney, 1992), supporting its construct validity with *r* = .45 and *r* = .31, respectively (Ponterotto *et al.*, 1998). Sum scores were calculated per participant. Higher scores indicate more positive attitudes and higher awareness. Reliability for the measure was α = .77.

### Data analysis

Analysing patterns of missing values indicated that more than 5% of the values were missing completely at random (MCAR) with χ^2^(1,220) = 1267.158, *p* = .170. Missing data were handled using pairwise deletions, as this method produces consistent and hence relatively unbiased estimates of the parameters when the data are MCAR (Allison, 2009). Checking the Mahalanobis distance using both sum scores and subscale scores from our measures indicated one multivariate outlier in our data (*df* = 8, α = .05), which was excluded from our sample.

To confirm the factor structure of the items, we retained from the Culturally Responsive Teaching Practices measure (based on Siwatu, 2007), we performed a factor analysis using the remaining sample. The value of Kaiser–Meyer–Olkin Measure of Sampling Adequacy (KMO) was .78, indicating that the strength of the relationships among items was high, and Bartlett’s test of sphericity was significant, χ^2^(190) = 644.521, *p* < .001. The data hence met the assumptions of factor analysis.

The factor analyses were performed using the maximum‐likelihood extraction method. An Oblimin rotation was used as factors were expected to be correlated. We first discovered the factor structure with an exploratory factor analysis, χ^2^(100) = 100.774, *p* = .459, and also examined a three‐factor solution, χ^2^(133) = 166.962, *p* = .025. However, in line with our conceptual categorization, the two‐factor solution fit our data the best, χ^2^(151) = 217.508, *p* < .001. The first factor had an eigenvalue of 5.338 and accounted for 26.7% of the variance in the data. Factor two had an eigenvalue of 2.106 and accounted for further 10.6% of the variance (see Appendix for the factor loadings).

In addition, we investigated whether there were any differences between groups of teachers with different ethnic identities regarding the main variables in our study. A one‐way MANOVA was performed with teachers’ self‐identified ethnic background (only Dutch, Dutch and another, only another) as the grouping variable, and their perspective taking, multicultural attitudes, and CRT as the variables to be compared. We did not find a significant difference on these variables based on ethnic background, *F*(8, 204) = .611, *p* = .606; Wilk's Λ = .940, partial η^2^ = .03 (see Table 1). Subsequently, participants who indicated another affiliation than Dutch (e.g., Turkish) or an additional ethnic affiliation to Dutch (e.g., Moroccan–Dutch) were grouped together to form one group for easier interpretation of our analysis results.

**Table 1:** MANOVA results for teachers grouped by their ethnic affiliation

	SS	*Df*	Mean square	*F*	Sig.	η^2^
Perspective taking	28.032	2	14.016	1.200	.305	.022
Multicultural attitudes	62.052	2	31.026	0.580	.562	.011
CRT: Culturally sensitive teaching	17.285	2	8.643	0.236	.790	.004
CRT: Socially sensitive teaching	11.048	2	5.524	0.520	.596	.010

Note

CRT = Culturally responsive teaching.

As we considered two predictor variables in order to explain values of two dependent variables (i.e., the two components extracted from CRT: culturally sensitive teaching and socially sensitive teaching), we used multivariate multiple regression to analyse our data. This approach is equivalent to performing separate univariate regressions independently for each dependent variable. However, the current analytical approach does not assume that the responses are independent from each other and do account for the correlations between the dependent variables (Johnson & Wichern, 2015). Type 3 sums of squares method was used to estimate the effects of predictors on the dependent variables after controlling for all the other variables in the model.

## Results

Table 2 presents descriptive statistics and zero‐order correlations among the variables. Teachers’ Background Qualities were not related to any of the outcome variables. The Concentration of Ethnically Minoritized Students in teachers’ classrooms, on the other hand, was related to teachers’ Attitudes, Perspective Taking Abilities, and their Culturally Sensitive Teaching. Teachers who reported more positive Multicultural Attitudes, higher Perspective Taking Abilities, and more frequent Culturally Responsive Teaching worked in schools that had higher Concentration of Minoritized Students.

**Table 2:** Descriptive statistics and zero‐order intercorrelations

	Mean	*SD*	1	2	3	4	5	6	7	8
1. Gender	–	–	1							
2. Ethnic background	–	–	.048	1						
3. Years of teaching	14.50	10.50	−.008	.076	1					
4. Minoritized student concentration	33.35 (100)	31.53	−.086	−.145	.099	1				
5. IRI: Perspective taking	24.77 (35)	3.75	.060	−.045	.201*	.284**	1			
6. Multicultural attitudes	71.73 (100)	7.39	−.065	.056	−.107	.289**	.235*	1		
7. CRT: Culturally sensitive teaching	38.07 (55)	3.25	−.044	.064	.183	.345**	.329**	.429**	1	
8. CRT: Socially sensitive teaching	38.37 (45)	6.01	−.009	.006	.087	.090	.243**	.227*	.425**	1

Note

The highest possible scores are indicated in parentheses.

Years of teaching is presented in years, which was strongly correlated with teachers’ age (*r* = .92, *p* < .01).

Minoritized student concentration in classrooms was strongly correlated with minoritized student concentration in schools (*r* = .88, *p* < .01). Hence, teachers’ age and their schools’ minoritized student concentration are not presented in this table.

*p < .05; ^**^ *p *< .01.

**Figure 1:**
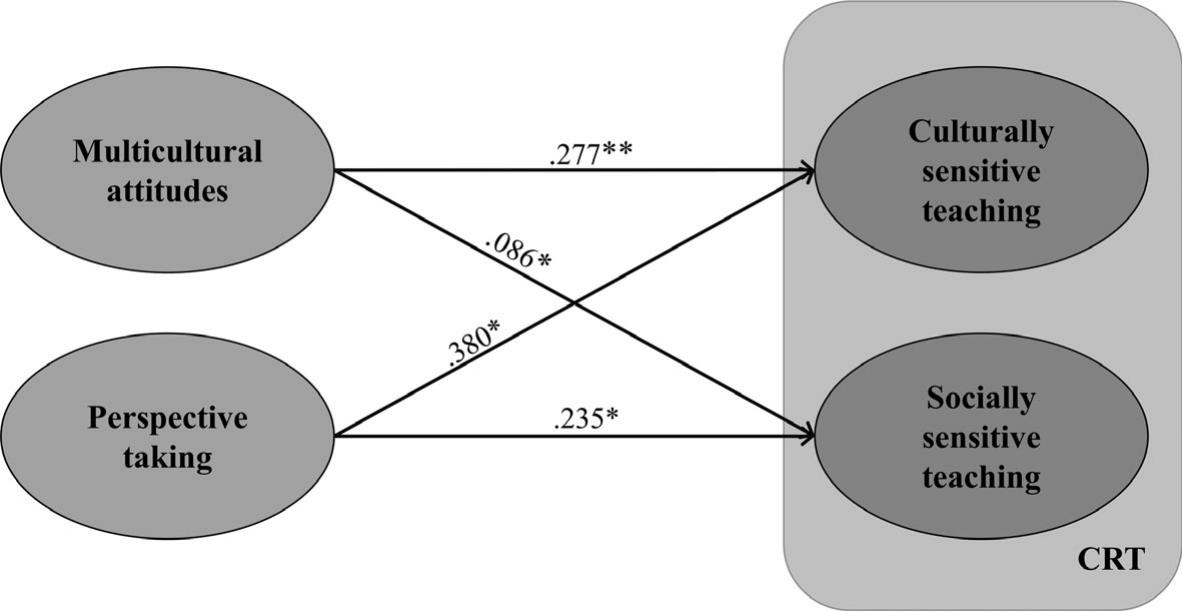
The multivariate multiple regression model. The regression coefficients are unstandardized (the measurement scale is the same for all variables). *p < .05; **p < .01. Model *R* ^2^ = .28.

In order to test our hypotheses that teachers’ Perspective Taking Abilities and Multicultural Attitudes are uniquely associated with Culturally Responsive Teaching Practices, we conducted a multivariate multiple regression analysis with Perspective Taking and Multicultural Attitudes as predictor variables, and their Culturally Sensitive Teaching and Socially Sensitive Teaching as the dependent variables, while we controlled for their classroom’s Ethnically Minoritized Student Concentration. The results of the analysis are presented in Figure 1.

Teachers’ more positive Multicultural Attitudes and higher Perspective Taking Abilities were significantly associated with engaging more in both the Culturally and Socially Sensitive aspects of Culturally Responsive Teaching. For both predictors, the relationship was stronger for the Culturally Sensitive compared to the Socially Sensitive Teaching component. Further, Perspective Taking, compared to Multicultural Attitudes, was a stronger predictor of both components.

## Discussion

The current study investigated teachers’ perspective taking abilities and their multicultural attitudes in relation to their self‐reported CRT practices. In doing so, we sought evidence in support of teacher qualities that have previously been suggested to be essential for CRT (Cooper, 2004; Grant & Asimeng‐Boahene, 2006; McAllister & Irvine, 2002; Nieto, 2004; Robins *et al.*, 2006; for a review see Rychly & Graves, 2012).

Our findings supported both hypotheses. Teachers who had better perspective taking abilities and more positive multicultural attitudes, reported to engage in CRT more frequently. Interestingly, both multicultural attitudes and perspective taking abilities better predicted culturally sensitive compared to socially sensitive teaching. Culturally sensitive teaching seems to be associated with practices that require greater willingness, effort, and ability to understand individual differences that relate to cultural elements. Socially sensitive teaching on the other hand seems to tap individual differences between students that are not necessarily due to cultural elements. Teacher qualities related to taking another persons’ perspective and being aware of diversity of experiences may thus support teachers’ attempts to effectively navigate through these differences.

Another important finding was that perspective taking was a stronger predictor for both components of CRT than multicultural attitudes were. One explanation for this finding could be that when reporting on their perspective taking abilities, teachers reflected relatively more on distinct cognitive processes in comparison with their attitudes, awareness, and beliefs, which are harder to recognize.

Finally, our results showed that teachers who reported more positive multicultural attitudes and better perspective taking abilities were appointed in schools with a higher concentration of ethnically minoritized students. This can be explained in two ways. These teachers might have actively chosen to teach in or did not drop out of schools/classrooms with higher minoritized student concentrations, because they feel more comfortable with dealing with diversity than their colleagues (Thijs & Verkuyten, 2014). Alternatively, teaching in rather diverse environments may have resulted in more positive multicultural attitudes and a stronger motivation to take others’ perspectives in teachers, due to an increased exposure to a diverse student body (Allport, 1954). Regardless, the finding that these teachers engage more frequently in the culturally and socially sensitive teaching aspects of CRT signals that perspective taking abilities and positive multicultural attitudes are both desirable teacher qualities for good teaching practices. Moreover, in line with previous research that showed that inducing perspective taking was effective in improving attitudes towards stigmatized groups such as the homeless (Batson *et al.*, 1997) and ethnic and racial minoritized groups (Finlay & Stephan, 2000), our results also showed that teachers who had better perspective taking abilities reported to have more positive multicultural attitudes.

### Practical importance

Teachers’ perspective taking abilities and multicultural attitudes seem critical for negotiating the complexities of diversity in classrooms. These qualities enable teachers to better align their teaching to their students’ needs. Our findings are promising for these qualities are malleable and thus can be improved inasmuch as teachers build on top of their existing knowledge on their students’ values, beliefs, communities, personal lives, and experiences.

Along these lines, Warren (2018) recommended three specific professional learning experiences that could further teachers’ perspective taking abilities. First, the author recommended teachers to get exposed to texts written on and by culturally and linguistically diverse populations in order to better recognize, determine, and scrutinize examples of institutionalized oppression. Second, the author recommended teachers to participate in the social worlds and realities of individuals from cultural communities that differ from their own. Such experiences should induce changes in teachers’ awareness, attitudes, beliefs, and values about cultural differences. Third, the author postulated that these experiences must be accompanied by critical dialogue with colleagues on a regular basis. Introspection on emotional, behavioural, and cognitive reactions towards students and their families should form the basis of these dialogues.

Thus, similar to perspective taking abilities, meaningful direct contact with people from diverse backgrounds (Allport, 1954), and opportunities to reflect on how culture shapes our values, beliefs, biases, and behaviours have been shown to improve attitudes and awareness (Case, 2007). Therefore, teacher education experiences similar to that recommended by Warren (2018) can be introduced to teacher education and professional development programmes. This would support teachers’ capacities to become more effective in teaching a diverse student body. Importantly, our results suggest that strengthening these capacities would not only improve the culturally sensitive teaching aspects of CRT but also teaching in a socially sensitive manner to student needs in general. As such, strengthening these capacities would benefit all students regardless of their backgrounds. These findings signal the significance of incorporating especially perspective taking experiences and exercises into teacher education and professional development programmes.

### Limitations and directions for future research

This study also has some limitations. First, although teachers’ own experiences and self‐knowledge are important sources of information, self‐reports are also subject to social desirability and self‐enhancing biases. The anonymity provided by online data collection, compared to other methods such as observations and interviews, helps mitigate this limitation. Yet, individuals may not be fully aware of their own biases, which may obstruct the accuracy of their self‐reports (McDonald, 2008). Future research may therefore include information from multiple informants to test the accuracy of these self‐report findings. For instance, the current study measured the willingness and tendency of teachers to take the perspective of others. Whether this is also reflected in their actual perspective taking in the classroom, however, was not investigated. 

Second, our measures were quantitative in nature because we aimed to find quantitative support for results from previous mainly qualitative studies. Future studies could include multiple assessment methods, which could contribute to the methodological robustness in measuring complex constructs similar to the ones used in our study. We should note, however, that the measures we used (e.g., the IRI) have been validated in the past in numerous studies, and have also been shown to be predictive of behavioural measures (Bonfils, Lysaker, Minor, & Salyers, 2017; Gini, Albiero, Benelli, & Altoè, 2007; Hawk *et al.*, 2013).

Third, the actual CRT practices of teachers were beyond the scope of this study. It is important that prospective studies investigate what CRT practices entail and how they differ for teachers with higher perspective taking abilities and more positive multicultural attitudes compared to their counterparts who are rather less skilled and whose attitudes are less positive. ‘The Culturally Responsive Instruction Observation Protocol’ (Powell, Cantrell, Malo‐Juvera, & Correll, 2016), providing a comprehensive operationalization of CRT around seven different elements, can be used in combination with self‐report measures to determine the extent of CRT implementation.

Finally, our study focused on the Dutch educational system and therefore we excluded items from the original (English) CRT measure that did not apply to the Dutch context (see Siwatu, 2007). Similar to any study of school context, some caution is therefore warranted with generalizing the results of this study to other settings. Moreover, we cannot exclude the possibility that teachers who are more positive on diversity matters were more likely to respond to our social media advertisement for recruiting participants. However, it should be noted that this type of research is almost always subject to selection bias, regardless of the recruitment method (Forgasz, Tan, Leder, & McLeod, 2018). That being said, with the increasing use of social network sites for participant recruitment, research on the representativeness of such samples has also increased. A recent study (Zhang *et al.*, 2018) compared results from separate surveys that included participants who were recruited using Facebook, who were independently recruited by a reputable survey research firm, and who were recruited by the American Community Survey, participation of which is required by law in the United States. The authors’ analyses yielded identical outcomes for the surveys regardless of their recruitment method. We are therefore confident that our recruitment method did not compromise the representativeness of our sample and the generalizability of our results.

Despite the limitations, our research supplements the literature with important first insights in a field that is under‐researched. Our results showed that positive attitudes and awareness about diversity, and perspective taking abilities are related to increases in cultural and social sensitivity in teaching. Hence, strengthening these capacities can improve the educational position of students with a migration history, as well as benefit their peers without any history of migration.

## Conflicts of interest

All authors declare no conflict of interest.

## Culturally responsive teaching practices

Conceptually, from the Culturally Responsive Teaching Practices measure (based on Siwatu, 2007), we retained items that fell under two categories, namely (1) culturally sensitive teaching and (2) socially sensitive teaching. In order to verify this categorization, we performed a factor analysis with two factors. The items and their factor loadings can be found in Table A1 below.

**Table A1:** Culturally responsive teaching: item selection and reduction

Retained Items		Factor loadings	
Loaded on culturally sensitive teaching factor (Factor 1)		Factor 1	Factor 2
CRT_5	Identify aspects in which the school culture (for example, values, norms, and practices) differs from the home culture of my students.	.652	−.021
CRT_12	Establish community between students when my class exists of students from various backgrounds.	.505	.239
CRT_13	Use the cultural background of my students to make learning meaningful.	.712	−.077
CRT_16	Obtain information regarding the cultural background of my students.	.592	.081
CRT_19	Design a classroom environment with attributes that represent a variety of cultures.	.478	−.041
CRT_27	Revise educational materials to improve its’ representation of cultural groups.	.664	−.021
CRT_28	Critically study the curriculum in order to determining whether it does or does not strengthen negative cultural stereotypes.	.358	.014
CRT_30	Design tasks in the classroom in a way that helps improve the understanding of students studying Dutch.	.521	−.072
CRT_31	Communicate with the parents of students studying Dutch about their child’s achievements.	.418	.198
CRT_35	Make use of examples that are relatable for students from culturally different backgrounds.	.700	−.073
CRT_37	Obtain information concerning my students’ academic interests.	.298	.249
CRT_38	Make use of my students’ interests to make learning meaningful to them.	.363	.236

Loaded on Socially Sensitive Teaching Factor (Factor 2)			
CRT_1	Adjust instructions to cater to the needs of my students.	−.054	.644
CRT_2	Obtain information regarding the academic strengths of my students.	−.112	.565
CRT_3	Assess whether my students rather work alone or in a group.	−.023	.284
CRT_7	Judge my students’ learning using various kinds of tests.	.016	.406
CRT_21	Obtain information regarding my students’ academic weaknesses.	−.011	.634
CRT_26	Help students establish positive relationships with their classmates.	.080	.463
CRT_34	Use a learning preference survey to obtain information on how my students prefer to learn.	.200	.397
CRT_40	Develop education according to my students’ developmental needs.	.135	.538
Excluded Items			
CRT_4	Assess whether my students are comfortable with competing with other students.	–	–
CRT_6	Implement strategies to minimize the effects of the mismatch between my students’ home culture and the school culture.	–	–
CRT_6	Implement strategies to minimize the effects of the mismatch between my students’ home culture and the school culture.	–	–
CRT_8	Obtain information regarding the home life of my students.	–	–
CRT_9	Establish a feeling of trust with my students.	–	–
CRT_10	Establish positive relationships between home and school.	–	–
CRT_11	Employ a variety of educational methods.	–	–
CRT_14	Use my students’ common knowledge to help them understand new information.	–	–
CRT_15	Identify how the way in which students communicate at home can differ from the school’s norms.	–	–
CRT_17	Teach students about their cultures’ contributions to science.	–	–
CRT_18	Greet students studying Dutch with a phrase from their mother tongue.	–	–
CRT_20	Establish a personal relationship with my students.	–	–
CRT_22	Praise students studying Dutch for their achievements, using a phrase in their mother tongue.	–	–
CRT_23	Identify ways in which standardized tests can be prejudiced against linguistically different students.	–	–
CRT_24	Communicate with parents regarding the progress of their child’s education.	–	–
CRT_25	Structure parent–teacher conferences in a way in which this meeting is not intimidating to parents.	–	–
CRT_29	Develop a lesson, which shows how other cultural groups have made use of mathematics.	–	–
CRT_33	Identify ways in which standardized tests can be prejudiced against culturally different students.	–	–
CRT_36	Explain new concepts using examples from my students’ daily lives.	–	–
CRT_39	Implement cooperative learning activities for students who prefer to work in groups.	–	–

Note

The second part of each item’s name represents the original item number within culturally responsive teaching measure (same as in Siwatu, 2007).

CRT = culturally responsive teaching.
